# Facile synthesis and strongly microstructure-dependent electrochemical properties of graphene/manganese dioxide composites for supercapacitors

**DOI:** 10.1186/1556-276X-9-490

**Published:** 2014-09-13

**Authors:** Caiyun Zhang, Xiaohong Zhu, Zhongxing Wang, Ping Sun, Yinjuan Ren, Jiliang Zhu, Jianguo Zhu, Dingquan Xiao

**Affiliations:** 1 Department of Materials Science, Sichuan University, Chengdu 610064, People's Republic of China

**Keywords:** Graphene, Manganese dioxide, Composite, Microstructure, Electrochemical properties, Supercapacitors

## Abstract

Graphene has attracted much attention since it was firstly stripped from graphite by two physicists in 2004, and the supercapacitor based on graphene has obtained wide attention and much investment as well. For practical applications of graphene-based supercapacitors, however, there are still many challenges to solve, for instance, to simplify the technological process, to lower the fabrication cost, and to improve the electrochemical performance. In this work, graphene/MnO_2_ composites are prepared by a microwave sintering method, and we report here a relatively simple method for the supercapacitor packaging, i.e., dipping Ni-foam into a graphene/MnO_2_ composite solution directly for a period of time to coat the active material on a current collector. It is found that the microwave reaction time has a significant effect on the microstructure of graphene/MnO_2_ composites, and consequently, the electrochemical properties of the supercapacitors based on graphene/MnO_2_ composites are strongly microstructure dependent. An appropriately longer microwave reaction time, namely, 15 min, facilitates a very dense and homogeneous microstructure of the graphene/MnO_2_ composites, and thus, excellent electrochemical performance is achieved in the supercapacitor device, including a high specific capacitance of 296 F/g and a high capacitance retention of 93% after 3,000 times of charging/discharging cycles.

**PACS:**

81.05.ue; 78.67.Sc; 88.80.fh

## Background

In recent years, with the deterioration of the environment and the scarcity of natural resources, more and more researchers have turned their attention to the field of energy. Supercapacitor, serving as a novel energy storage device, is one of the mostly focused topics. The materials for supercapacitors that have been intensively studied so far can be divided into three groups: transition metal oxides, carbon materials, and conductive polymers [1]. In the first group, MnO_2_ and RuO_2_ are two typical materials, and they are usually used for the fast and reversible redox reactions since the pseudocapacitance generated from the faradaic redox reactions is helpful for a remarkable increase in the specific capacitance of supercapacitors. However, metal oxides usually have a high electrical resistance, thereby leading to a low power density, and the high cost of RuO_2_ also limits its wide applications. For the second group, they are usually used in double-layer capacitors because of their electrochemical stability and high accessible surface area. For the third group, polyaniline and polypyrrole [2-5] for instance, they show high specific capacitance whether in aqueous or in nonaqueous electrolytes. However, the conductive polymers become unstable with extended lifetimes of charging/discharging. This may reduce severely the initial performance for supercapacitors [6]. Due to the unique character in each supercapacitor material, it is significant for us to develop a composite material for supercapacitors, which takes advantage of both double-layer capacitors and the faradaic pseudocapacitors. Graphene/MnO_2_ composite is such an alternative material.

Owing to its high specific surface area, remarkable chemical stability, and superior electron mobility [1,7,8], graphene can greatly improve the performance of the supercapacitors when it is used as an electrode material. Meanwhile, for manganese dioxide, there are many advantages, such as low cost, high energy density, natural abundance, and being environmentally friendly [9-11]. Furthermore, as mentioned above, the redox reaction itself in MnO_2_ can form a pseudocapacitance, thereby increasing the capacitance of the capacitor. To prepare graphene more simply and more safely, and finally to make the mass production come true, various methods have been developed [12], including mechanical exfoliation [13], chemical vapor deposition [14,15], pressurized oxidization/reduction [16], epitaxial growth [17], and chemical oxidation/reduction [18-20]. In this work, graphene was firstly prepared by the chemical oxidation/reduction method, and then manganese dioxide-modified graphene composites were prepared by a simple microwave method [21]. The redox reaction under microwave irradiation between carbon and KMnO_4_ in a pH-neutral solution is expressed as follows [22]:

(1) $$4{{\mathrm{MnO}}_{4}}^{-}+3C+H_{2}O\;\Leftrightarrow 4{\mathrm{MnO}}_{2}+{{\mathrm{CO}}_{3}}^{2-}+2{{\mathrm{HCO}}_{3}}^{-}\text{.}$$

It is noteworthy that a simple method was used here for the supercapacitor packaging, i.e., dipping Ni-foam into a graphene/MnO_2_ composite solution directly for a period of time to coat the active material on a current collector. Moreover, the process-structure-property relationships were systematically investigated for the graphene/MnO_2_ composites. Interestingly, it was found that the microwave reaction time has a significant effect on the microstructure of graphene/MnO_2_ composites and the electrochemical properties of the supercapacitors based on graphene/MnO_2_ composites are therefore strongly microstructure dependent.

## Methods

### Synthesis: graphene and graphene/MnO_2_ composite

Graphite oxide (GO) was synthesized firstly from natural graphite according to a modified Hummers method [23]. A GO solution that displays a brown dispersion was subsequently prepared. For purification, the mixture was successively washed with 5% HCl and deionized water for several times to completely remove residual salts and acids. Once the filter cake was dried in an electric thermostatic drying oven at 40°C, the graphene oxide powders were obtained. After that, the graphene oxide powder (640 mg) was dispersed in 600 ml deionized water and then sonicated until it was well distributed. Twenty milliliters hydrazine hydrate was added into the suspension, and the suspension was then kept at 90°C for 24 h [1,23]. Finally, the suspension was filtered and washed several times with deionized water and alcohol, and then dried at 50°C for 12 h in a vacuum oven.

Graphene/MnO_2_ composites were prepared by a redox reaction between graphene and potassium permanganate under microwave irradiation [21]. In the first step, 100 ml of graphene water suspension (1.65 mg/ml) was subjected to ultrasonic vibration for 1 h. Then KMnO_4_ powder (0.95 g) was added into the graphene suspension and stirred for about 10 min. Subsequently, the resulted suspension was heated using a household microwave oven (Midea, Foshan, China, 2,450 MHz, 700 W) for several minutes, and then cooled to room temperature naturally. Finally, the black deposit was filtered and washed several times with distilled water and alcohol, and then dried at 80°C for 6 h in a vacuum oven. Different microwave reaction times, namely, 5, 10, and 15 min, were selectively used to study its effect on the microstructure and the electrochemical properties of the graphene/MnO_2_ composites. Due to the lab condition restriction and, more importantly, in order to avoid the overflowing of the reaction solution, we did not extend the microwave reaction time further.

### Characterization

The crystallographic structures of the graphene/MnO_2_ composites were measured by X-ray diffraction (XRD; DX-1000, Dandong Fangyuan Instrument Co., Ltd., Dandong, China) using Cu Kα radiation (*λ* = 0.154056 nm). The microstructure was characterized by scanning electron microscopy (SEM; Hitachi S-4800, Hitachi, Ltd., Chiyoda-ku, Japan, operated at 30 kV) and transmission electron microscopy (TEM; JEOL JEM-2100 F, JEOL, Ltd., Akishima-shi, Japan, operated at 200 kV). Note that the samples were dispersed in alcohol and dropped on a holey copper grid for TEM observations. The electrochemical measurements (cyclic voltammetry, galvanostatic charge/discharge, and electrochemical impedance spectroscopy) were measured by using an electrochemical station (CHI 660E, CH Instruments, Inc., Austin, TX, USA) with a two-electrode system, which consists of two identical working electrodes in 6 M KOH alkaline electrolytes.

### Electrochemical measurements

The fabrication of working electrodes was carried out in the following way. Briefly, the materials, including graphene/MnO_2_ composite, carbon black, and polytetrafluoroethylene (PTFE), were mixed in a mass ratio of 75:20:5 and dispersed in ethanol [21]. Then the nickel foam substrate in the form of small rounds was dipped directly into the as-prepared suspension for several minutes and subsequently dried at 80°C for 12 h in a vacuum oven. Since the nickel foam is easy to be oxidized and its surface may contain oil, the nickel foam was cleaned sequentially by acetone, deionized water, diluted hydrochloric acid, deionized water, and ethanol before it was used. Taking out the dried Ni-foam with active material coated and exerting 10-MPa pressure by a table press, we got the electrode slices for use. After dipping the electrode slices into a 6 M KOH alkaline electrolyte solution for 24 h, we assembled the button-type supercapacitor for tests.

## Results and discussion

**Figure 1:**
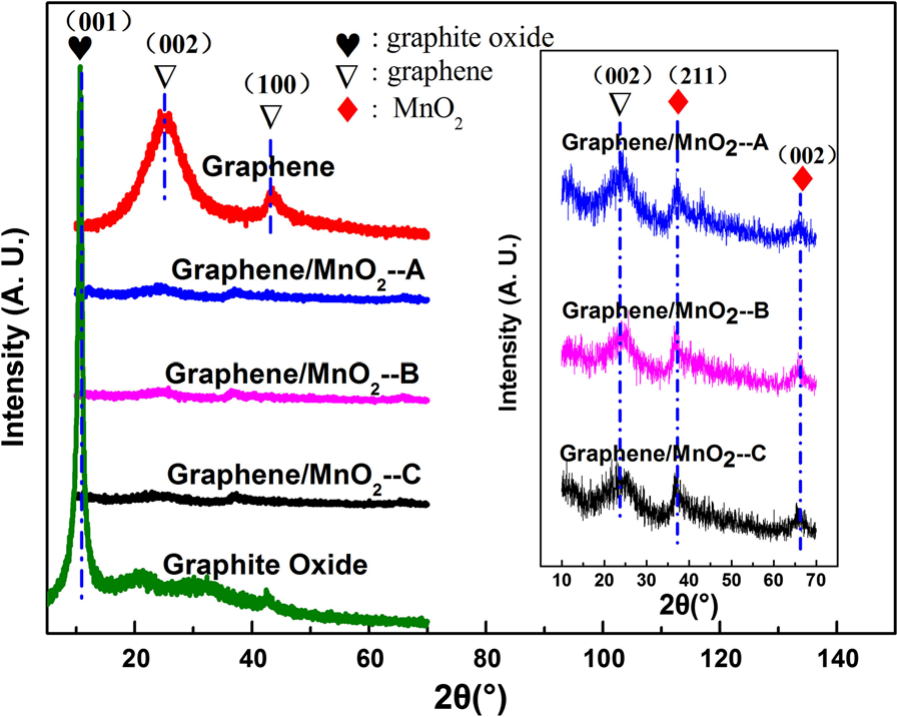
**XRD patterns of the graphene, graphite oxide, and graphene/MnO**_**2 **_**composites.** Note that the three composite samples are represented as graphene/MnO_2_--A (5 min), graphene/MnO_2_--B (10 min), and graphene/MnO_2_--C (15 min). The inset shows the refined XRD patterns for the three samples with different microwave reaction times (5, 10, and 15 min).

**Figure 2:**
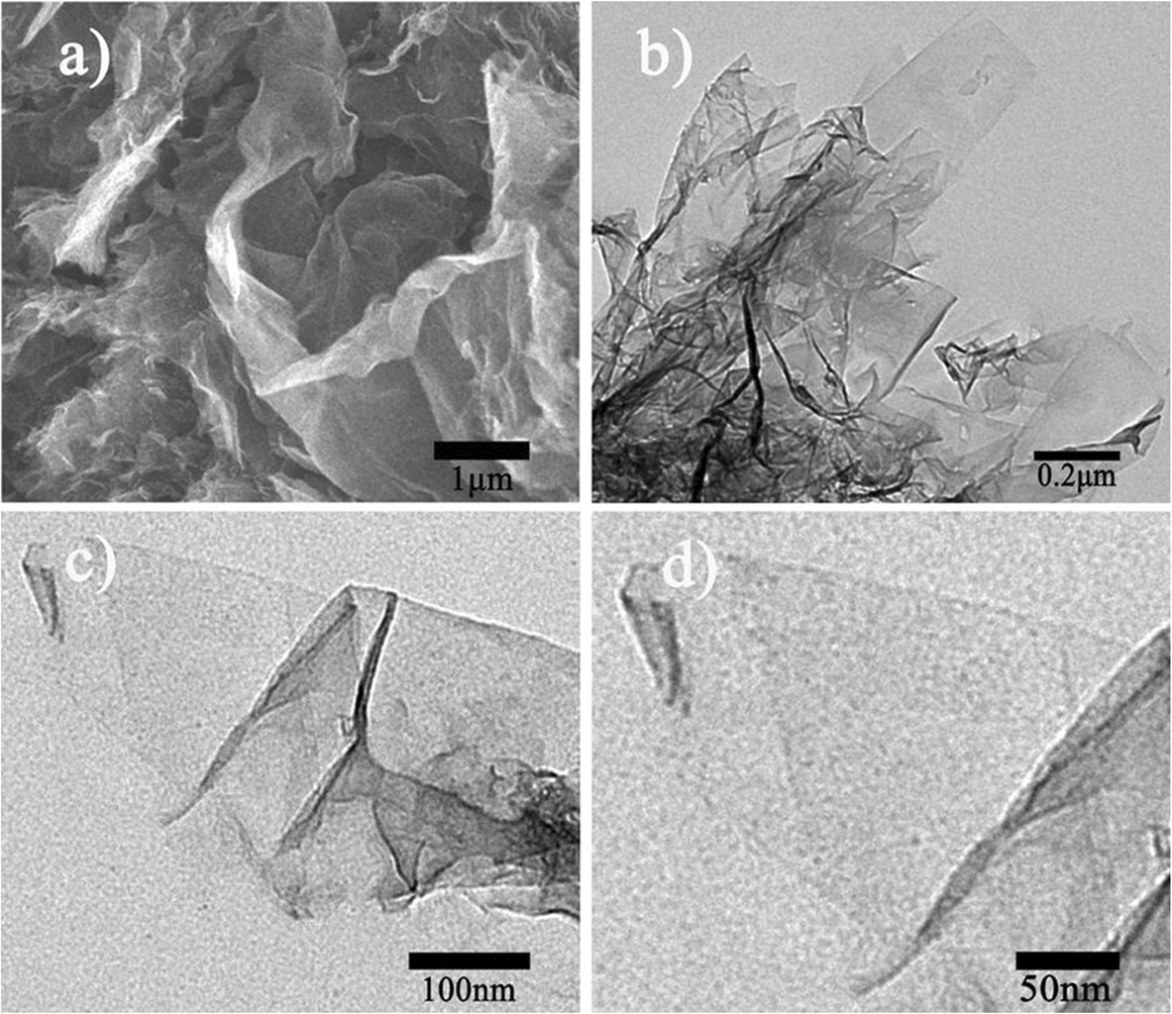
**SEM and TEM images of the graphene prepared in this work. (a)** SEM micrograph for the graphene, chemically reduced from graphene oxide with hydrazine monohydrate. **(b-d)** TEM micrographs for the graphene.

Figure 1 shows the XRD patterns for graphene, graphite oxide, and graphene/MnO_2_ composites with different microwave reaction times (5, 10, and 15 min). It can be seen that the as-prepared graphene shows a weak and broad diffraction peak at 2*θ* = 23°, corresponding to the diffraction of the (002) plane. For the three graphene/MnO_2_ composite samples, their XRD patterns are almost the same, exhibiting a weak and broad peak of the (002) plane centered at 2*θ* = 23° (indexed for graphene) and two weak peaks indexed for α-MnO_2_ at 2*θ* = 37° (211) and 2*θ* = 66° (002) [24]. The peak at 2*θ* ~ 43°, corresponding to the (100) crystal plane of graphene, cannot be observed after the deposition of MnO_2_, indicating that the surfaces of graphene were fully covered by nanoscale MnO_2_ and thus a lower degree of graphitization was induced, which was similarly reported by Yan et al. [21]. Just for XRD patterns, as the microwave time increases, there is no distinct difference between these three samples. But it can be speculated from the XRD patterns that the three graphene/MnO_2_ composite samples have similar chemical compositions. To know more about the influence of microwave reaction time on the graphene/MnO_2_ composites, their microstructure and electrochemical properties have been systematically investigated, as will be discussed below.Figure 2 gives microstructural information for the graphene prepared in this work. Figure 2a is a SEM micrograph of the graphene after chemical reduction from graphene oxide with hydrazine monohydrate. It is interesting to note that the graphene shows a wrinkled paper-like morphology. Figure 2b,c,d shows the TEM micrographs with different magnifications for the graphene. As we can see, there exists a single-layer sheet on the edge of the graphene; however, there are also multilayers overlapped in the central part of the sample due to the strong van der Waals interactions.

**Figure 3:**
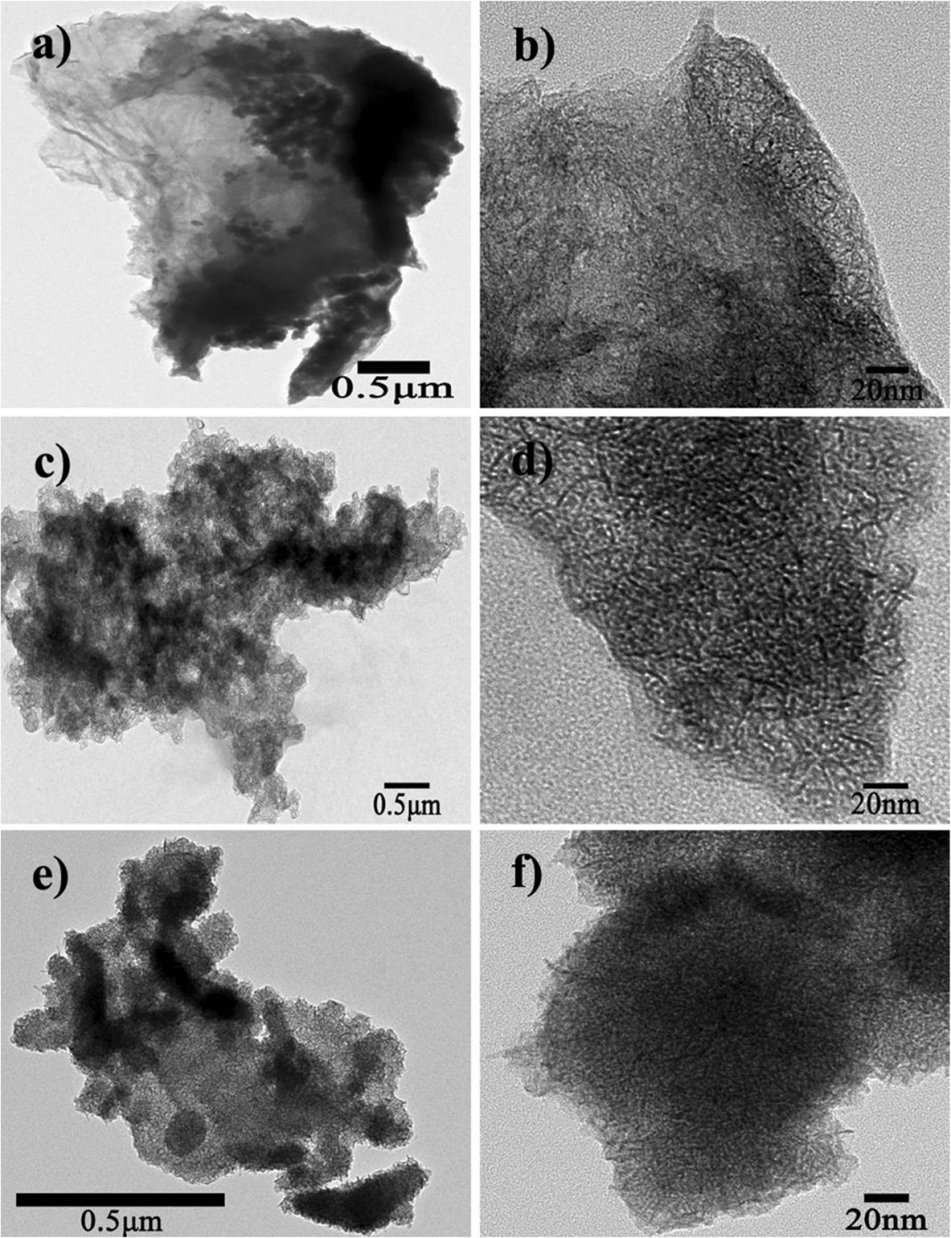
**TEM micrographs of the graphene/MnO**_**2 **_**composites.** From top to bottom, the images correspond respectively to 5-, 10-, and 15-min microwave reaction times. The left panels **(a, c, e)** are low-magnification images and the right panels **(b, d, f)** are high-magnification TEM images of the composites.

Figure 3 shows TEM micrographs of the graphene/MnO_2_ composites prepared with different microwave reaction times. Judging from Figure 3, we conclude that, as the microwave reaction time increases, the graphene cannot be seen directly in the micrographs due to high-density coating of MnO_2_[1]. However, it is shown in the low-magnification TEM images (Figure 3a,c,e) that the MnO_2_ nanoneedles grew almost on the whole graphene surface. Especially from the high-magnification TEM images (Figure 3b,d,f), we can see clearly that the MnO_2_ coating on the graphene sheet looks like needles, and they integrate closely with each other. On the other hand, by contrast with Figure 2, the presence of MnO_2_, to some extent, lowers the stacking of graphene sheets due to van der Waals interactions [24], which can markedly enlarge the specific surface area of the composite. Furthermore, the needlelike MnO_2_ is conductive to the diffusion of ions, leading to a reduced diffusion resistance and thus an improved electrochemical response of the composite electrodes. It should be pointed out that, as the microwave reaction time increases, the redox reaction between graphene and potassium permanganate can proceed more sufficiently, and MnO_2_ can deposit on graphene sheets much more uniformly as well, thereby facilitating a denser and more homogeneous microstructure in the graphene/MnO_2_ composites. Accordingly, the sample with a 15-min microwave reaction time exhibits the best morphology, for which MnO_2_ was relatively well distributed on the whole graphene sheet.

To characterize the electrochemical behavior of the button-type supercapacitor based on graphene/MnO_2_ composites, we have carried out cyclic voltammetry (CV), galvanostatic charge/discharge (CD), and electrochemical impedance spectroscopy (EIS) tests using a two-electrode system. To avoid complex steps, we adopted a simple method of dipping directly the as-prepared Ni-foam into the suspension of graphene/MnO_2_ composite, carbon black, and PTFE to prepare electrode slices. This method is simple to operate, and the active materials are not easy to fall off once they have been coated on Ni-foam. Furthermore, the active materials are relatively well distributed. However, there is also a disadvantage, that is, it is hard to control the mass of the active materials. Hence, we cannot get the difference in specific capacitance directly just by looking at the CV and CD diagrams since the mass of the active materials is different in those three samples. Nonetheless, the mass of the active materials can be calculated out by comparatively weighing the Ni-foam before and after coating the active materials, and the specific capacitance can be therefore obtained for each supercapacitor device.

**Figure 4:**
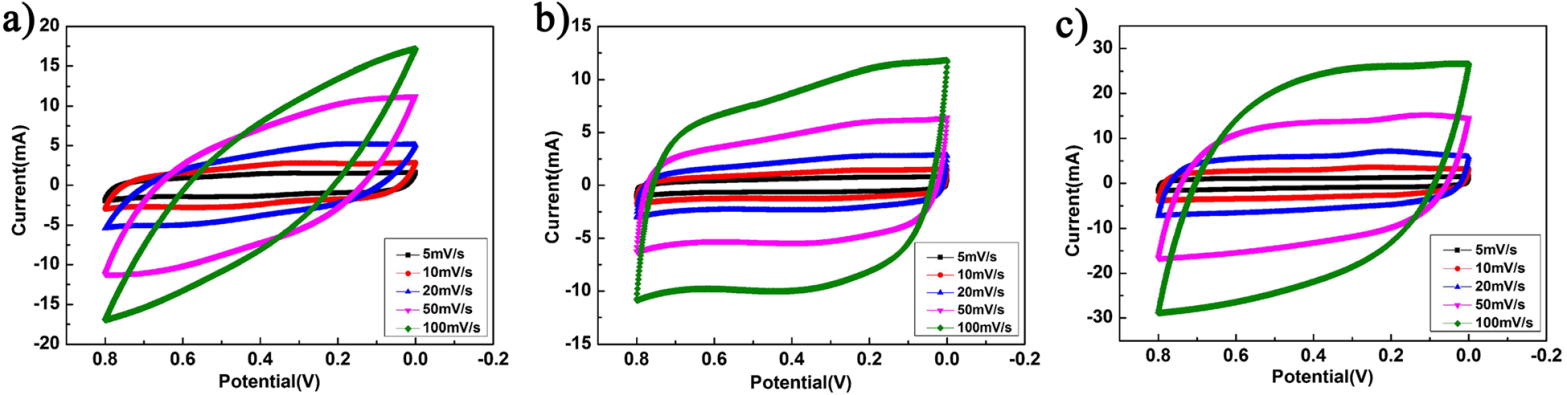
**CV curves of the graphene/MnO**_**2 **_**composite electrodes.** The curves were measured at different scan rates of 5, 10, 20, 50, and 100 mV/s (from inner to outer). From **(a)** to **(c)**, the microwave reaction times are 5, 10, and 15 min, respectively. Note that the mass of the active material is similar for **(a)** and **(c)**, which is, however, almost twice as that for **(b)**.

Figure 4 presents the cyclic voltammetric curves of the supercapacitors based on graphene/MnO_2_ composites with various scan rates in the range of 5 to 100 mV/s within a 0 to 0.8-V voltage window. It is well known that the more similar the shape of the CV cycles of a supercapacitor to a rectangle, the lower the contact resistance of the supercapacitor [25,26]. As the microwave reaction time increases, especially from 5 to 10 min, the CV curves of our devices are getting closer to a rectangle at various scan rates, even at the high scan rate of 100 mV/s, indicating an excellent capacitive behavior and a low contact resistance in the supercapacitor [27,28]. The difference among the three devices can be well explained in terms of their different microstructural features, as already shown in Figure 3. With the increase in the microwave reaction time, the needlelike MnO_2_ gets firstly a little larger and then smaller again, but always tends to distribute more uniformly on almost the whole graphene sheet, and thus the relatively less stacking of graphene might be achieved as well. This could increase the specific surface area, thereby enhancing the electrical conductivity of the device.

**Figure 5:**
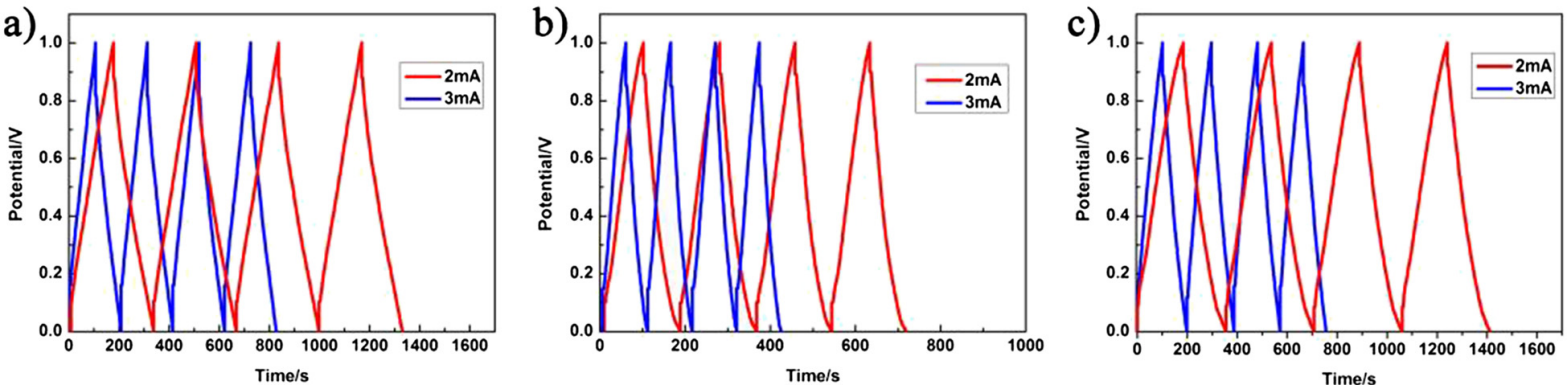
**Galvanostatic charge/discharge curves of the supercapacitors based on graphene/MnO**_**2 **_**composite electrodes with different microwave reaction times. (a)** 5 min, **(b)** 10 min, and **(c)** 15 min. For each sample, the curves were measured at charging current of 2 and 3 mA, respectively.

Figure 5 shows galvanostatic charge/discharge curves of the supercapacitors with different microwave reaction times for the synthesis of graphene/MnO_2_ composites. All the CD curves are linear and symmetric in the voltage range 0 to 1 V, indicating very good electrochemical properties of the graphene/MnO_2_ composite electrodes [29]. Using the experimental data displayed in the galvanostatic charge/discharge curves, the specific capacitance has been calculated from the two-electrode cell-specific capacitance by the formula given below:

(2) $$c_{m}=4\frac{I\times t}{m\times V}\text{,}$$

where *I* is the change/discharge current, *t* is the discharging time, *m* is the mass of the active material of two electrodes, and *V* is the voltage window after the deduction of the IR drop. Accordingly, as the microwave reaction time increases from 5 to 15 min, the specific capacitance at a charging current of 2 mA reaches 246, 260, and 296 F/g, respectively. As aforementioned, though the mass of the active material in each device is different, we can get the specific capacitance values similarly through calculating the data in CV curves. The enhancement of specific capacitance with increasing the microwave reaction time is believed to stem from the improved microstructure, as mentioned above. The results obtained in this work are comparable to or even higher than those reported in the literature for similar graphene-based material systems, where the specific capacitance was reported to be about 200 to 300 F/g [30-32].

**Figure 6:**
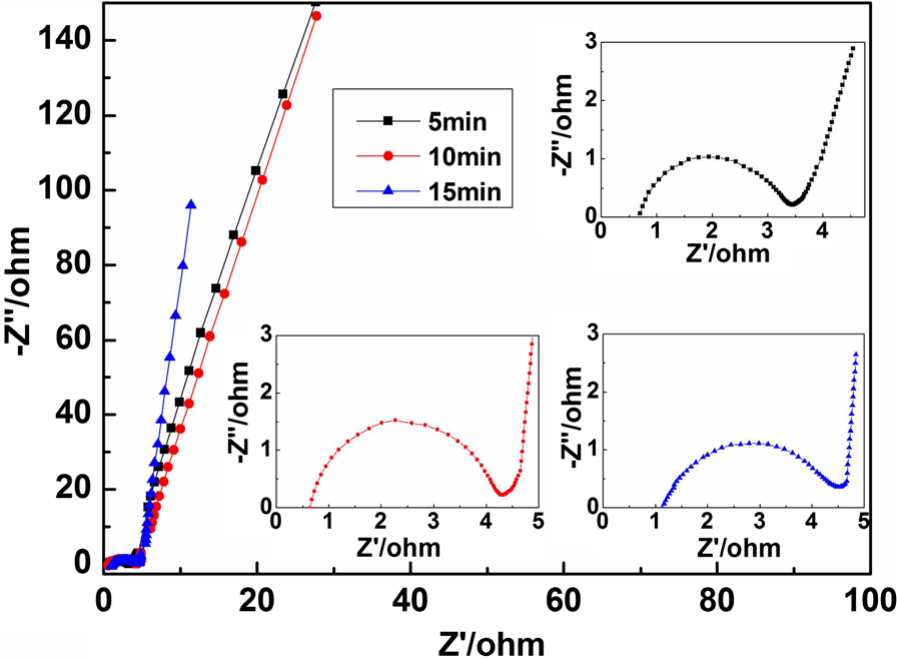
**Impedance spectroscopies of the supercapacitors based on graphene/MnO**_**2 **_**composites with different microwave reaction times.** All the curves displayed here were measured in the frequency range of 100 kHz to 0.01 Hz. *Z*′ is the real impedance and *Z*″ is the imaginary impedance. The insets show an enlarged scale for the impedance spectroscopy images.

Electrochemical impedance spectroscopy is a very significant measure to evaluate the quality of supercapacitors. Figure 6 shows the impedance curves of the supercapacitors based on graphene/MnO_2_ electrodes with different microwave reaction times, measured in a 6 M KOH alkaline electrolyte solution. The horizontal axis intercepts at high frequency in the Nyquist plots for the devices with microwave times 5, 10, and 15 min are, respectively, 0.70, 0.65, and 1.13 Ω, indicating that with the change in microwave reaction time, the electronic resistance (*R*_s_), including the ionic resistance of the electrolyte, the intrinsic resistance of the substrate, and the contact resistance at the interface of the active material/current collector [33], changes inconspicuously. The radius of the semicircle represents the charge transfer resistance (*R*_ct_) at the electrode/electrolyte interface [34-36]. The shorter 45° portion of the curve demonstrates the faster ion diffusion in the electrolyte to the electrode interface for the sample with a microwave reaction time of 15 min [28,37-39]. At low frequency, the slope of the curve reveals the quality of a capacitor. The more vertical the curve is, the better the ion transport is and the higher performance the supercapacitor has. As shown in Figure 6, at a low-frequency region, the device with a microwave reaction time of 15 min demonstrates a near 90° angle, indicative of a great capacitive characteristic [39].

**Figure 7:**
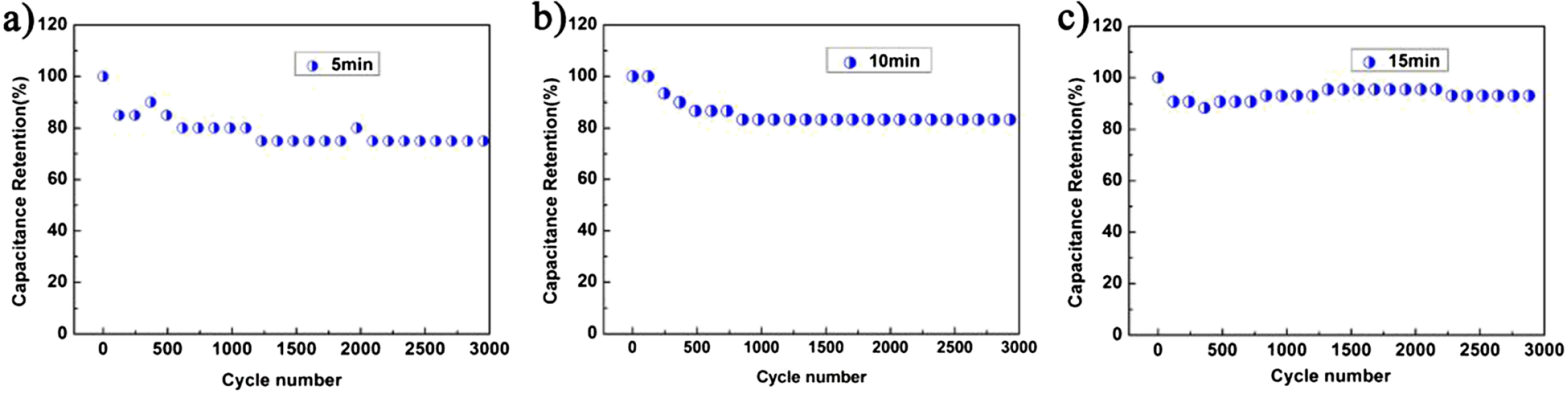
**Cycling performance of the supercapacitors based on graphene/MnO**_**2 **_**composites.** The capacitance retention is roughly 75%, 83%, and 93% after 3,000 cycles of charging and discharging at a current density of 2 mA for the microwave reaction times **(a)** 5 min, **(b)** 10 min, and **(c)** 15 min, respectively.

Another critically important factor to evaluate the quality of a supercapacitor is the cycling stability. It is shown in Figure 7 that the capacitance retention increases remarkably as the microwave reaction time increases from 5 to 15 min. Compared to 75% (5 min sample) and 83% (10 min sample), the capacitance retention of the sample with a microwave reaction time of 15 min still remains as high as 93% after 3,000 times of charging/discharging cycles, demonstrating an excellent electrochemical stability for the graphene/MnO_2_ composite with improved microstructure.

## Conclusions

High-quality graphene/MnO_2_ composites have been prepared by a relatively simple microwave sintering method, and a simple method, i.e., dipping Ni-foam into a graphene/MnO_2_ composite solution directly for a period of time to coat the active material on a current collector, has been proposed for the supercapacitor packaging. It is demonstrated that the microwave reaction time has a significant effect on the microstructure of graphene/MnO_2_ composites and the electrochemical properties of the supercapacitors based on graphene/MnO_2_ composites are strongly microstructure dependent. An appropriately longer microwave reaction time, namely, 15 min, facilitates a very dense and homogeneous microstructure of the graphene/MnO_2_ composites, and thus, excellent electrochemical performance has been achieved in the supercapacitor device based on the graphene/MnO_2_ composite, comprising a high specific capacitance of 296 F/g and a high capacitance retention of 93% after 3,000 times of charging/discharging cycles. The results obtained in this work pave the way for facile synthesis and optimization of graphene-based composite materials for practical supercapacitor applications.

## Competing interests

The authors declare that they have no competing interests.

## Authors' contributions

XZ designed the study. CZ, ZW, and PS performed the preparation and characterization of materials and supercapacitor devices. YR, JZ (Jiliang), JZ (Jianguo), and DX contributed to the data analysis and discussion. CZ and XZ analyzed the data completely and co-wrote the manuscript. All authors reviewed the manuscript. All authors read and approved the final manuscript.

## Authors' information

CZ is a postgraduate student at Sichuan University (SCU). XZ has a PhD degree and is a professor at SCU. ZW and PS have a master's degree. YR is a postgraduate student at SCU. JZ (Jiliang) has a PhD degree and is a professor at SCU. JZ (Jianguo) has a PhD degree and is a professor and head of a school at SCU. DX is a professor and head of a research laboratory at SCU.
